# Respiratory Viral Shedding in Healthcare Workers Reinfected with SARS-CoV-2, Brazil, 2020

**DOI:** 10.3201/eid2706.210558

**Published:** 2021-06

**Authors:** Mariene R. Amorim, William M. Souza, Antonio C.G. Barros, Daniel A. Toledo-Teixeira, Karina Bispo dos-Santos, Camila L. Simeoni, Pierina L. Parise, Aline Vieira, Julia Forato, Ingra M. Claro, Luciana S. Mofatto, Priscila P. Barbosa, Natalia S. Brunetti, Emerson S.S. França, Gisele A. Pedroso, Barbara F.N. Carvalho, Tania R. Zaccariotto, Kamila C.S. Krywacz, André S. Vieira, Marcelo A. Mori, Alessandro S. Farias, Maria H.P. Pavan, Luís Felipe Bachur, Luís G.O. Cardoso, Fernando R. Spilki, Ester C. Sabino, Nuno R. Faria, Magnun N.N. Santos, Rodrigo Angerami, Patricia A.F. Leme, Angelica Schreiber, Maria L. Moretti, Fabiana Granja, José Luiz Proenca-Modena

**Affiliations:** University of Campinas, Campinas, Brazil (M.R. Amorim, A.C.G. Barros Jr., D.A. Toledo-Teixeira, K. Bispo-dos-Santos, C.L. Simeoni, P.L. Parise, A. Vieira, J. Forato, L.S. Mofatto, P.P. Barbosa, N.S. Brunetti, E.S.S. França, G.A. Pedroso, B.F.N. Carvalho, T.R. Zaccariotto, K.C.S. Krywacz, A.S. Vieira, M.A. Mori, A.S. Farias, M.H.P. Pavan, L.F. Bachur, L.G.O. Cardoso, M.N.N. Santos, R. Angerami, P.A.F. Leme, A. Schreiber, M.L. Moretti, F. Granja, J.L. Proenca-Modena);; University of São Paulo, São Paulo, Brazil (W.M. Souza, I.M. Claro, E.C. Sabino, N.R. Faria); Feevale University, Novo Hamburgo, Brazil (F.R. Spilki);; University of Oxford, Oxford, UK (N.R. Faria);; Imperial College London, London, UK (N.R. Faria);; Campinas Department of Public Health Surveillance, Campinas (R. Angerami);; Federal University of Roraima, Boa Vista, Brazil (F. Granja)

**Keywords:** SARS-CoV-2, COVID-19, reinfection, healthcare workers, virus isolation, MinIon Sequencing, D614G mutation, respiratory infections, severe acute respiratory syndrome coronavirus 2, 2019 novel coronavirus disease, coronavirus disease, zoonoses, viruses, coronavirus, Brazil, viral shedding, variants

## Abstract

We documented 4 cases of severe acute respiratory syndrome coronavirus 2 reinfection by non–variant of concern strains among healthcare workers in Campinas, Brazil. We isolated infectious particles from nasopharyngeal secretions during both infection episodes. Improved and continued protection measures are necessary to mitigate the risk for reinfection among healthcare workers.

Coronavirus disease (COVID-19) is caused by severe acute respiratory syndrome coronavirus 2 (SARS-CoV-2), which emerged in Wuhan, China, in late 2019. As of April 8, 2021, COVID-19 has affected >132 million persons and caused >2.87 million deaths around the world (https://covid19.who.int). Whether the immune response elicited by an initial infection protects against reinfection is uncertain. The Pan American Health Organization provisionally defines reinfection as a positive SARS-CoV-2 test result >45 days after initial infection, given that other infections and prolonged shedding of SARS-CoV-2 or viral RNA have been ruled out (*1*). Healthcare workers (HCWs) are consistently exposed to SARS-CoV-2 and are therefore susceptible to reinfection.

We investigated 4 cases of SARS-CoV-2 reinfection among HCWs at the Hospital das Clínicas da Unicamp, a tertiary public hospital at the University of Campinas (Campinas, Brazil). This study was approved by the Research Ethical Committee of the University of Campinas (approval no. CAAE-31170720.3.0000.5404). The 4 HCWs, consisting of 3 nurses and 1 staff member, were women with an average age of 44 years (range 40–61 years) (Figure 1, panel A). For the initial infections, symptom onset ranged from April 5–May 10, 2020, and lasted 10–23 days. We identified SARS-CoV-2 RNA in nasopharyngeal swab samples using real-time quantitative reverse transcription PCR (qRT-PCR) 2–4 days after symptom onset (*2*). All 4 HCWs had mild COVID-19 signs and symptoms and recovered (Table). After signs and symptoms resolved, the HCWs tested negative by qRT-PCR, Elecsys Anti-SARS-CoV-2 (Roche Diagnostics, https://diagnostics.roche.com), or both. Reinfection, confirmed by a nucleic acid amplification test using the GeneFinder COVID-19 Plus RealAmp Kit (*3*), developed 55–170 days after symptom onset of the first infection. Signs and symptoms of reinfection lasted 9–23 days. Only 1 HCW had a concurrent condition (chronic bronchitis), and none were immunosuppressed. None required hospitalization during the initial or reinfection episodes (Table). After recovering from their initial infections, all HWCs continued to use the same types of personal protective equipment (i.e., disposable surgical masks, gloves, gowns, and goggles) in accordance with recommendations from the Ministry of Health of Brazil (https://coronavirus.saude.gov.br/saude-e-seguranca-do-trabalhador-epi).

**Table:** Characteristics of healthcare workers with severe acute respiratory syndrome coronavirus 2 reinfections, Brazil, 2020*

Characteristic	Healthcare worker			
	1	2	3	4
Underlying conditions	None	Chronic bronchitis	None	None
Hospitalized	No	No	No	No
Symptoms				
First infection	Fever, headache, chills, sneezing, coryza, and myalgia	Headache, cough, myalgia, odynophagy, coryza, diarrhea, and ageusia	Nasal congestion, coryza, cough, ageusia	Fever, headache, myalgia, coryza, dry cough, vomiting, and malaise
Second infection	Headache, nasal congestion, odynophagia, ageusia, and anosmia	Cough, myalgia, odynophagia, anosmia, and diarrhea	Odynophagia, sneezing, coryza, diarrhea, ageusia, and anosmia	Odynophagia, dry cough, myalgia, malaise, coryza, and headache
Cycle threshold values				
First infection†	E gene: 35.24; N gene: 40.12	E gene: 31.8	E gene: 35.15	E gene: 34.80; RdRp gene: 39.86
Second infection‡	E gene: 31.14; N gene: 31.3; RdRp gene: 32.58	E gene: 20.45; N gene: 20.52; RdRp gene: 22.65	E gene: 26.04; N gene: 26.88; RdRp gene: 28.40	E gene: 23.72; N gene: 23.48; RdRp gene: 25.67
Time between symptom onsets, d	55	170	131	148

*E gene, envelope gene; N gene, nucleoprotein gene; RdRp gene, RNA dependent RNA polymerase gene. †Real-time quantitative reverse transcription PCR selective for the envelope gene (*2*). ‡Nucleic acid amplification test using the GeneFinder COVID-19 Plus RealAmp Kit (OSANG Healthcare Co. Ltd., http://www.osanghc.com) (*3*).

To assess whether infectious SARS-CoV-2 particles were shed in nasopharyngeal secretions during both COVID-19 episodes, we conducted viral isolation in Vero cells (ATCC no. CCL-81) (W.M. de Souza, unpub. data, https://dx.doi.org/10.2139/ssrn.3793486) (Appendix). We inoculated Vero cells with isolated SARS-CoV-2 virions from nasopharyngeal swab samples collected during the first and second infections; we observed a cytopathic effect 3–4 days after inoculation. On day 4, we obtained cell culture supernatant by centrifugation and conducted qRT-PCR selective for the envelope gene to confirm the presence of SARS-CoV-2 RNA; we found the supernatants had 2.8 × 10^2^–1.4 × 10^10^ RNA copies/mL (*2*). We confirmed viral isolation by the increased number of RNA copies per milliliter and the decreased cycle threshold values after passage into Vero cells. The isolation of SARS-CoV-2 shows that nasopharyngeal swab samples contained infectious particles during both COVID-19 episodes.

SARS-CoV-2 variants of concern (VOCs; i.e., lineages B.1.1.7, B.1.351, and P.1.), and particularly their mutations in the spike protein, have been associated with reinfection (*4*,*5*). To investigate this association, we sequenced SARS-CoV-2 genomes from samples or isolates in this study using the ARTIC version 3 protocol (https://artic.network/ncov-2019) with MinION sequencing (Oxford Nanopore Technologies, https://nanoporetech.com). We obtained sequences with 66%–99% genome coverage (mean depth >20-fold) for 3 of 4 HCWs (Appendix). We submitted the sequences to GISAID (https://www.gisaid.org; accession nos. EPI_ISL_1511399, EPI_ISL_1511603, EPI_ISL_1511641, and EPI_ISL_1511644). We used the Pangolin COVID-19 Lineage Assigner tool (*6*) to classify samples as members of lineages B.1.1.28 (n = 3) and B.1.1.33 (n = 1); 3 of these samples were taken during the reinfection episodes of HCWs 1, 2, and 4 and 1 during the first episode of HCW 1 (Appendix). These lineages have circulated in Brazil since early March 2020 (*7*) and have not been associated with reinfection or long-term infection. In addition, we found the D614G mutation in the spike protein in samples from both episodes of HCW 1 and the second episode of HCW 2. The D614G mutation has been associated with enhanced viral replication in the upper respiratory tract and increased susceptibility of the virus to neutralization by antibodies (*8*). In addition, we found the V1176F mutation in the spike protein in samples from both episodes of HCW 1 and the second episode of HCW 4; however, the effects of this mutation remain unclear. None of the genomes had the mutations in spike proteins described in 3 recent VOCs (https://cov-lineages.org). Other cases of SARS-CoV-2 reinfection by strains without mutations in the spike protein were documented in India; those infections were associated with lineages B.1.1.8 and B.1.1.29 (*9*). Our results provide additional evidence of SARS-CoV-2 reinfection by non-VOC strains.

In conclusion, we report cases of SARS-CoV-2 reinfection among HCWs. We observed the shedding of infectious viral particles during both infection episodes of each HCW. Hence, the continuation of protective measures, as well as efforts to monitor, track exposures, and identify areas at high risk for infection, are critical to reducing SARS-CoV-2 reinfection, especially among HCWs.

**Figure:**
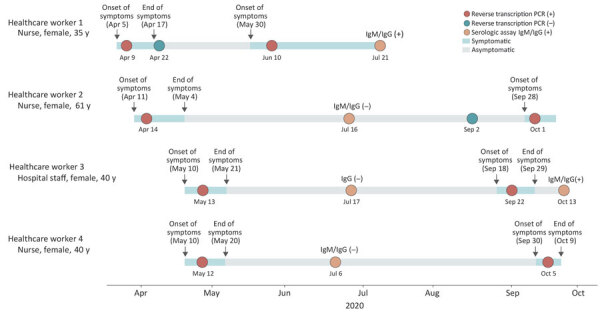
Timeline of severe acute respiratory syndrome coronavirus 2 reinfections (SARS-CoV-2) among healthcare workers, Brazil, 2020. (+), positive; (–), negative.

Appendix Additional information for study of respiratory viral shedding in healthcare workers reinfected with SARS-CoV-2, Brazil, 2020.
