# Supplementary material for: Respiratory Viral Shedding in Healthcare Workers Reinfected with SARS-CoV-2, Brazil, 2020
Source: Emerg Infect Dis. 2021 Jun;27(6):1737–40. doi: 10.3201/eid2706.210558 (PMC8153890; doi:10.3201/eid2706.210558)
Supplement: Appendix — Additional information for study of respiratory viral shedding in healthcare workers reinfected with SARS-CoV-2, Brazil, 2020. [file 21-0558-Techapp-s1.pdf]

Article DOI: https://doi.org/10.3201/eid2706.210558

Respiratory Viral Shedding in Healthcare Workers Reinfected with SARS-CoV-2, Brazil, 2020

Appendix

Materials and Methods

Sample Collection and Ethics

We used residual serum and nasopharyngeal swab samples collected at distinct time points corresponding to the period when patients had coronavirus disease (COVID-19) symptoms. Data, including age, sex, occupation, sample collection data, symptoms, and concurrent conditions, were collected from electronic health records (Appendix Table).

SARS-CoV-2 Diagnosis

Viral RNA was extracted using the Quick-RNA viral kit (Zymo Research, https://www.zymoresearch.com) following the manufacturer recommended procedures. The RNA from samples were tested for severe acute respiratory syndrome coronavirus 2 (SARS-CoV-2) by real-time quantitative reverse transcription PCR selective for the envelope gene and nucleic acid amplification test using the GeneFinder COVID-19 Plus RealAmp Kit (OSANG Healthcare Co. Ltd., http://www.osanghc.com) (Appendix Table) (*2*,*3*). In addition, the IgM and IgG antibodies against SARS-CoV-2 proteins were measured by Elecsys Anti-SARS-CoV-2 (Roche Diagnostics, https://diagnostics.roche.com), according to the manufacturer instructions.

Virus Isolation

Nasopharyngeal lavage samples were inoculated into Vero cells (CCL-81) for virus isolation using the method described previously (W.M. de Souza, upub. data, https://papers.ssrn.com/sol3/papers.cfm?abstract_id=3793486). Briefly, 5×10^5 cells/mL of Vero cells were plated in a T225 flask with Dulbecco’s Modified Eagle Medium (DMEM) containing 10% fetal bovine serum and 1% of 10,000 units of penicillin and 10 mg of streptomycin/mL solution (Sigma-Aldrich Inc., https://www.sigmaaldrich.com). Next, samples were thawed on ice, diluted 1:10 in DMEM, and centrifuged at 12.000 × g for 5 min

at 4°C. Samples were filtered using 0.22 μm syringe filters, and incubated on ice for 1 h with a solution of DMEM with 10,000 units of penicillin, 10 mg of streptomycin/mL, and 250 μg/mL amphotericin B (i.e., 1:1 ratio) (Sigma-Aldrich, Inc.) in a final dilution of 1:10. After incubation at 37°C for 1 h, the inoculum was removed and replaced with fresh culture medium. Cells were incubated at 37°C and observed for cytopathic effects daily up to 4 days. Supernatant was collected daily and viral replication was confirmed through the increase of cycle threshold value using real-time quantitative reverse transcription PCR (*2*). All experiments related to culture cells and viral replication were performed in the Biosafety Level 3 laboratory at the Emerging Viruses Laboratory of the University of Campinas, Campinas, Brazil.

SARS-CoV-2 Genome Sequencing and Analysis

SARS-CoV-2 genome sequencing was carried out using the ARTIC version 3 (https://artic.network/ncov-2019) protocol with MinION sequencing (Oxford Nanopore Technologies, https://nanoporetech.com). cDNA was synthesized using the extracted RNA with random hexamers and the Protoscript II First Strand cDNA synthesis Kit (New England Biolabs, https://www.neb.com). Then, we performed the SARS-CoV-2 whole-genome multiplex-PCR amplification using ARTIC network SARS-CoV-2 V3 primer scheme and Q5 High-Fidelity DNA polymerase (New England Biolabs). The PCR product was purified using AMPure XP magnetic beads (Beckman Coulter, Inc., https://www.beckmancoulter.com), according to manufacturer instructions. DNA was quantified using Qubit dsDNA High Sensitivity assay on the Qubit 3.0 (Thermo Fischer Scientific, https://www.thermofisher.com). To uniform sequencing, equimolar normalization of 10 ng per sample was performed followed by barcoding using the EXP-NBD104 Native Barcoding Kits (Oxford Nanopore Technologies). Then, barcoded samples were pooled followed by library preparation using the SQK-LSK109 Kit (Oxford Nanopore Technologies). Finally, Nanopore sequencing libraries were loaded onto an R9.4.1 flow-cell (Oxford Nanopore Technologies) and sequenced using MinKNOW version 20.10.3 (Oxford Nanopore Technologies). FAST5 files containing the raw signal data were basecalled, demultiplexed, and trimmed using Guppy version 4.4.1 (Oxford Nanopore Technologies). The reads were aligned against the reference genome Wuhan-Hu-1 (GenBank accession no. MN908947) using minimap2 version 2.17.r941 (https://github.com/lh3/minimap2) and converted to a sorted BAM file using SAMtools (http://samtools.sourceforge.net). Length filtering, quality test, primmer trimming, variant calling and consensus sequences were performed for each

barcode using guppyplex from ARTIC (https://artic.network/ncov-2019). Genome regions with a depth of <20-fold were represented with N characters. Tablet alignment viewer (version 1.19.09.03) (https://ics.hutton.ac.uk/tablet) was used to visualize the mapped sequence. Finally, the 2 genomes were uploaded to the CoV-GLUE online resource (J. Singer, unpub. data, https://www.preprints.org/manuscript/202006.0225/v1) for mutation determination. The sequences have been uploaded to GISAID under the accession nos. EPI_ISL_1511399, EPI_ISL_1511603, EPI_ISL_1511641, and EPI_ISL_1511644.

HCW 1 (first infection) EPI_ISL_1511641 (coverage = 99.4%)		
Gene	Nucleotide	Amino acid
nsp7	C12053T	L71F
nsp12	C14408T	P323L
nsp15	C20132T	A171V
S	A23403G	D614G
	G25088T	V1176F
N	G28881A,G28882A	R203K
	G28883C	G204R

HCW 1 (second infection) EPI_ISL_1511644 (coverage=97.0%)		
Gene	Nucleotide	Amino acid
nsp7	C12053T	L71F
nsp12	C14408T	P323L
nsp15	C20132T	A171V
S	A23403G	D614G
	G25088T	V1176F
N	G28881A,G28882A	R203K
	G28883C	G204R

HCW 2 (second infection) EPI_ISL_1511399 (coverage = 97.2%)		
Gene	Nucleotide	Amino acid
nsp2	C1601T	L266F
	G2447T	G548C
nsp6	G11317T	M115I
nsp12	C14408T	P323L
nsp14	G18255T	M72I
S	A23403G	D614G
	G23587C	Q675H
E	C26447T	S68F
M	G26763T	A81S
ORF 6	T27299C	I33T
N	G28881A, G28882A	R203K
	G28883C	G204R
	T29148C	I292T
	G29513T	A414S

HCW 4 (second infection) EPI_ISL_1511603 (coverage = 66.3%)		
Gene	Nucleotide	Amino acid
nsp2	A1682C	I293L
nsp6	C11036T	L22F
nsp12	C14408T	P323L
S	G25088T	V1176F
N	G28655C	D128H
	G28881A,G28882A	R203K
	G28883C	G204R

Appendix Figure. Severe acute respiratory syndrome coronavirus mutations in 3 of 4 reinfected HCWs, Brazil, 2020. Wuhan-Hu-1 (GenBank accession no. MN908947) used as reference sequence. E, envelope protein; HCW, healthcare worker; M, matrix protein; N, nucleoprotein; S, spike protein.
